# A machine learning approach for saddle height classification in cycling

**DOI:** 10.3389/fspor.2025.1607212

**Published:** 2025-09-17

**Authors:** Fangbo Bing, Guoxin Zhang, Linjuan Wei, Ming Zhang

**Affiliations:** ^1^ Department of Biomedical Engineering, Faculty of Engineering, The Hong Kong Polytechnic University, Hong Kong SAR, China; ^2^ Research Institute for Sports and Technology, The Hong Kong Polytechnic University, Hong Kong SAR, China

**Keywords:** cycling, joint angle, lower limb, machine learning, saddle height

## Abstract

**Background:**

Saddle height is an important factor in bike fitting because it correlates with cycling efficiency and the risk of injuries. Conventional approaches use anthropometric parameters and joint angles as references to calculate the optimal saddle height, such as the greater trochanter height and knee flexion angle. However, these methods fail to consider individual dynamic differences in cycling.

**Objective:**

This study proposed a machine learning (ML) model for calculating saddle height based on easily measured kinematic data.

**Method:**

In total, 16 subjects participated in riding tests at three saddle heights. The motion capture system recorded the trajectories of markers attached to their lower limbs. Features were calculated using the hip, knee, and ankle joint angles. The optimal feature set was selected using forward sequential feature selection. The accuracies of four ML models were compared using leave-one-subject-out cross-validation.

**Results:**

The optimal feature set contained 14 features related to the hip, knee, and ankle joint angles. The sagittal plane knee angle was the most sensitive to the saddle height, with a classification accuracy of 80%. The *k*-nearest neighbor model had the highest accuracy of 99.79% when using all the optimal features as inputs.

**Conclusion:**

The proposed model compensates for the lack of consideration in traditional methods of individual dynamic variations in cycling, providing a more objective tool for data-driven personalization in bike fitting.

## 1 Introduction

Cycling is becoming more and more popular. However, the number of overuse injuries related to cycling has also increased. Proper bike fitting is important to reduce the risk of injuries and increase cycling efficiency (1). Traditional bike fitting methods rely on static measurements, empirical rules, and subjective feedback from cyclists, which may not fully account for individual biomechanical variations or dynamic riding conditions. Saddle height is one of the most studied variables in bike fitting because it has a greater impact on the range of motion (ROM) of the lower limb joints and muscles than other variables, such as handlebar height and crank length (2). A change of 2% in saddle height can significantly alter lower limb kinematics, affecting the extension and flexion angles of the hip and knee joints and their ROMs (3). Changes in saddle height of more than 4% can cause changes in oxygen uptake and riding efficiency (2). Therefore, the lower limb joint angles are sensitive to alterations in saddle height. Previous studies have indicated that the saddle height should be set with a knee angle of 25°–35° when the crank is at the bottom dead center (BDC) (4, 5). However, static knee angles fail to match the dynamics of joint motion, especially at the 6 o'clock crank position, where the differences between the static and dynamic angles can reach 8.2 ± 5° (6). The peak joint loading during actual pedaling can reach two times the cyclist’s body weight, which is much higher than that in the static situation (7). Moreover, cyclists tend to adjust their kinematics (e.g., pelvic rotation, ankle dorsiflexion) to compensate for suboptimal saddle heights, masking the true biomechanical relationship in static measurements (8). The force–length–velocity relationships of muscles vary among cyclists and are not detectable in static measurements. Some equations have been proposed to determine saddle height based on anthropometric measurements and joint angles (9, 10). However, these methods have not been verified in a diverse group of cyclists and may not be applicable to certain female cyclists.

In recent years, machine learning (ML) has been used in sports science, offering data-driven insights and personalized solutions (11). Previous studies have demonstrated the advantages of ML in solving practical problems in biomechanics (12, 13). Compared with motion capture systems and instrumented sensors, ML can reduce the cost and duration related to the evaluation of sports performance. Several studies have applied ML to cycling, including using long-short memory neural networks to predict heart rate (14), pulmonary oxygen uptake (15), and cadence (16). Moreover, power output in riding has been estimated without measurement by a gradient boosting algorithm (17) and a tree-based model with random forest (18). The cycling efficiency index, which reflects the cycling state, can be predicted by artificial neural networks with recursive feature elimination based on the lower limb joint kinematics, power, cadence, and individual mass (19). Pedal force is essential in assessing pedaling efficiency, but it requires equipment to measure. A neural network model was used to predict radial and mediolateral pedal forces based on power, cadence, and crank angle (20). However, vertical force could not be predicted, and the accuracy of the mediolateral force needs to be improved. In addition, a variety of competitions can benefit from cycling route optimization and race rank prediction using various ML models (21).

Notwithstanding the promising applications of ML in cycling, there are several restrictions. The dataset of most ML models is from professional cyclists and cycling races, limiting their applicability to amateur cyclists. Several studies involved fewer than 10 participants, restricting the applicability of these ML models (16, 17, 22). In addition, current studies focus on physiological metrics such as heart rate and oxygen uptake and overlook biomechanical factors such as joint kinematics and bicycle configurations.

Proper saddle height is crucial for injury prevention and pedaling efficiency, but ML applications in cycling have not adequately explored this aspect. The purpose of this study was to develop an ML model that can accurately calculate saddle height based on lower limb joint angles during dynamic riding. Automatically distinguishing the appropriate saddle height will help cyclists optimize their riding posture for better performance and reduce joint stress to avoid overuse injuries.

## 2 Methods

**Figure 1:**
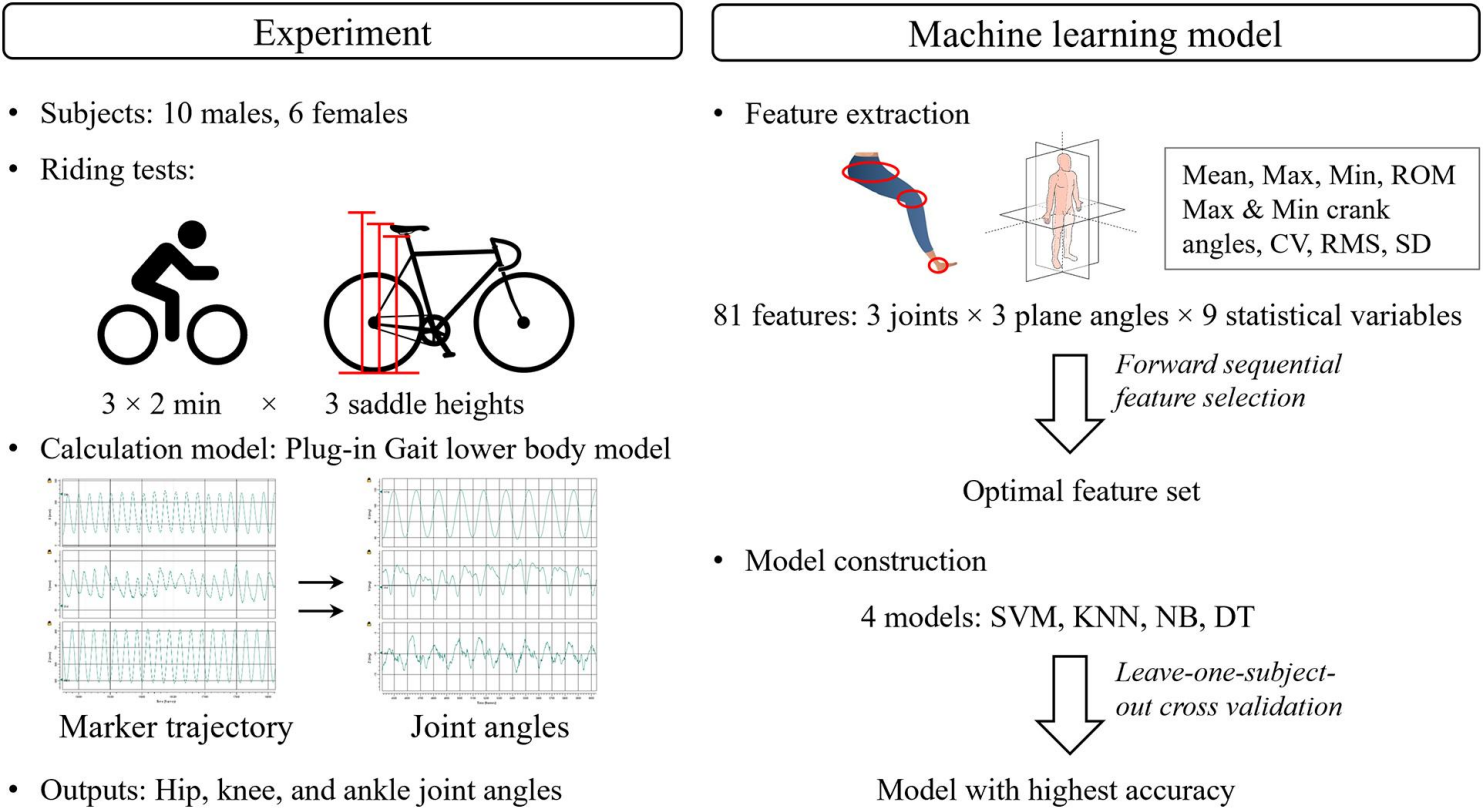
The framework of research. SVM, support vector machine; KNN, *k*-nearest neighbors; NB, Naïve Bayes; DT, decision trees; ROM, range of motion; CV, coefficient of variation; RMS, root mean square; SD, standard deviation.

This study consisted of two parts, as shown in Figure 1. In the cycling experiment part, subjects were recruited to perform riding tests at different saddle heights. The lower limb joint angles were calculated using Vicon Nexus 2.16. In the ML model development part, the model with the highest accuracy was constructed after comparing the performances of four ML models based on the selected optimal features.

### 2.1 Participants

The inclusion criteria for the participants were healthy individuals between 20 and 30 years old, with a BMI between 19 and 24 kg/m^2^ and a height of 165–180 cm for males or 155–175 cm for females. Furthermore, they were required to have reported riding more than once a week for longer than 30 min in daily life. In total, 16 amateur cyclists (10 males and 6 females, 24.64 ± 3.19 years, BMI of 21.34 ± 2.0 kg/m^2^) were recruited who reported that they had not been diagnosed with any musculoskeletal disease in the previous 6 months. All the participants signed informed consent forms after being informed about the experimental procedure and precautions. The experiment was approved by the university’s Human Subjects Ethics Sub-Committee (Number: HSEARS20220615001).

### 2.2 Experiment protocol

The subjects wore uniform, tight-fitting sportswear and their own sneakers, with sole thicknesses not exceeding 3.5 cm. The riding tests were conducted on a mountain bike on a smart training platform (Tacx NEO 2T, Garmin, USA). The bike configuration was uniform except for the saddle height, which was set to low, moderate, and high levels. According to a previous study, saddle heights that were 95% of an individual’s greater trochanter height (GTH) and 105% of their GTH were defined as low and high levels, respectively. A saddle height between 97% and 103% of one’s GTH was defined as the moderate level.

After warm-up exercises and test riding, the participants performed three 2-min rides at low, moderate, and high saddle height levels, respectively. They were given plenty of rest time between each ride to avoid fatigue. In total, 16 reflective markers were attached to the participants as required by the lower limb model in the motion capture system (Vicon Motion Analysis Inc., Oxford, UK), and were placed at the anterior and posterior superior iliac spine, and the thigh, knee, tibia, ankle, heel, and toes of the left and right lower limbs. The trajectories of these markers were recorded at 250 Hz by the system. The dynamic plug-in gait model was processed in Vicon Nexus 2.16 to calculate the kinematic results. The trajectory of the right ankle marker (RANK), which was placed on the lateral malleolus along an imaginary line that passed through the transmalleolar axis, and the joint angles of the hip, knee, and ankle of the right leg were output for further analysis.

### 2.3 Dataset

The trajectory of the RANK marker was first filtered by a zero-lag fourth-order low-pass filter with a cutoff frequency of 6 Hz. The interval between two adjacent *z*-coordinate maxima was defined as a pedaling cycle. Outlier data were excluded from the subsequent analysis. The lower limb joint angle data were divided according to the defined pedaling cycles. The synthetic minority oversampling technique was performed in Python 3.10 (Python Software Foundation, USA) to balance the number of datasets among the three groups. The number of resampled datasets was 72,354, with 24,118 in each saddle height category.

### 2.4 Feature extraction

**Figure 2:**
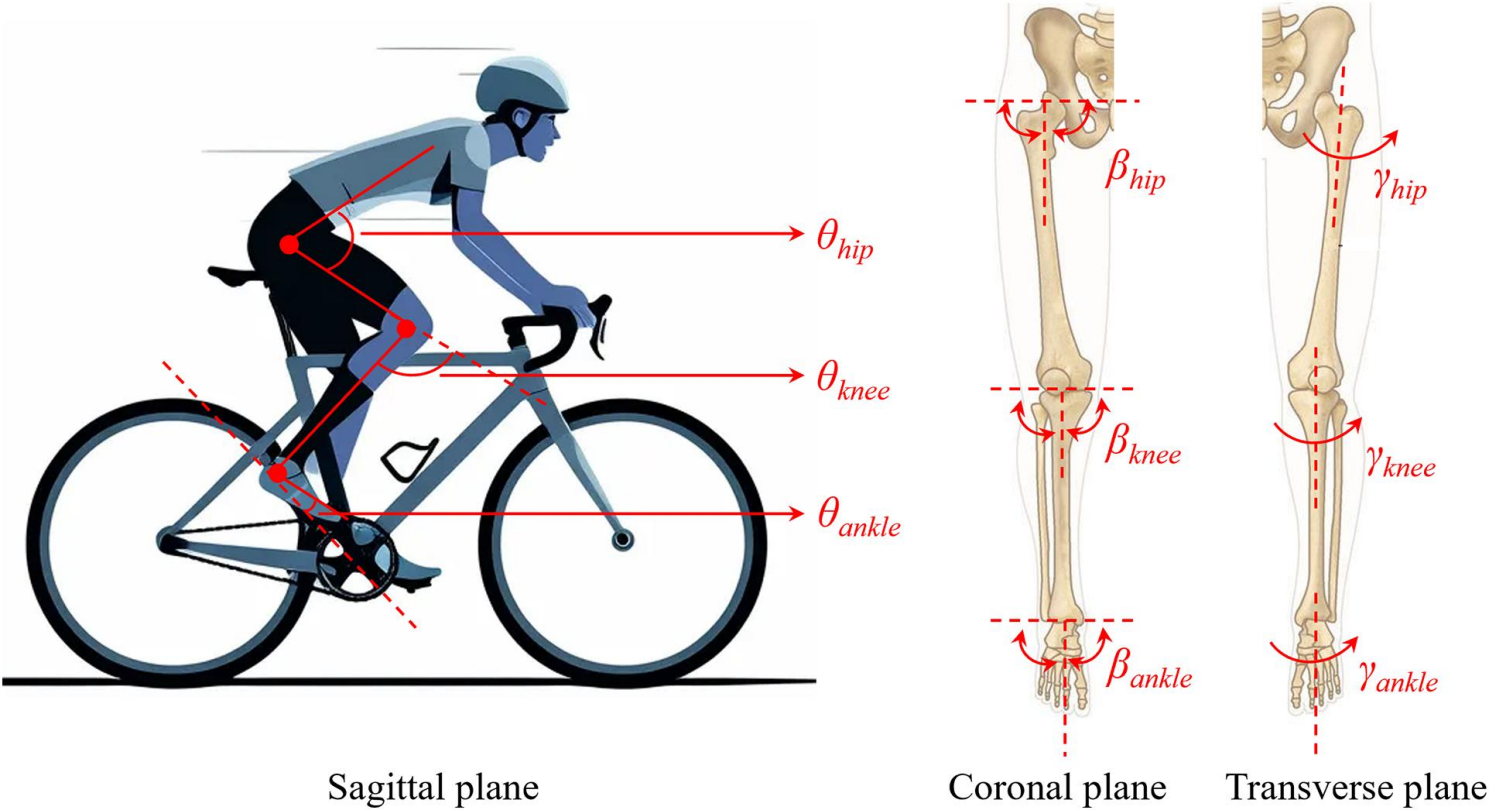
Diagram of the hip, knee, and ankle joint angles.

A series of features were extracted from the joint angles, including the maximum and minimum angles, the corresponding crank angle for the maximum and minimum angles (ranging from 0° to 360°), the root mean square (RMS) value, mean, standard deviation (SD), coefficient of variability (CV) (23), and the ROM (Figure 1). The hip, knee, and ankle joints each have three joint angles, namely the sagittal plane angle, coronal plane angle, and transverse plane angle (Figure 2). Therefore, 81 features (nine statistical types of features × three joints × three component angles) were extracted in every pedaling cycle, as summarized in Table 1. The features were normalized by their maximums. The final constructed feature array totaled 72,354 × 81. The label vector totaled 72,354 × 1, containing categories 1 (low saddle height), 2 (moderate saddle height), and 3 (high saddle height). The participant number vector totaled 72,354 × 1, containing numbers from 1 to 16 that were used to label the participant to which the features of each row belonged.

**Table 1:** Features extracted from joint angles.

Joint	Plane of angle	Features
Hip	Sagittal	$\theta_{\mathrm{hip},\mathrm{Max}}$, $\theta_{\mathrm{hip},\mathrm{Min}}$, $\theta_{\mathrm{hip},\mathrm{maxTiming}}$, $\theta_{\mathrm{hip},\mathrm{minTiming}}$, $\theta_{\mathrm{hip},\mathrm{RMS}}$, $\theta_{\mathrm{hip},\mathrm{Mean}}$, $\theta_{\mathrm{hip},\mathrm{SD}}$, $\theta_{\mathrm{hip},\mathrm{CV}}$, $\theta_{\mathrm{hip},\mathrm{ROM}}$
	Coronal	$\beta_{\mathrm{hip},\mathrm{Max}}$, $\beta_{\mathrm{hip},\mathrm{Min}}$, $\beta_{\mathrm{hip},\mathrm{maxTiming}}$, $\beta_{\mathrm{hip},\mathrm{minTiming}}$, $\beta_{\mathrm{hip},\mathrm{RMS}}$, $\beta_{\mathrm{hip},\mathrm{Mean}}$, $\beta_{\mathrm{hip},\mathrm{SD}}$, $\beta_{\mathrm{hip},\mathrm{CV}}$, $\beta_{\mathrm{hip},\mathrm{ROM}}$
	Transverse	$\gamma_{\mathrm{hip},\mathrm{Max}}$, $\gamma_{\mathrm{hip},\mathrm{Min}}$, $\gamma_{\mathrm{hip},\mathrm{maxTiming}}$, $\gamma_{\mathrm{hip},\mathrm{minTiming}}$, $\gamma_{\mathrm{hip},\mathrm{RMS}}$, $\gamma_{\mathrm{hip},\;\mathrm{Mean}}$, $\gamma_{\mathrm{hip},\mathrm{SD}}$, $\gamma_{\mathrm{hip},\mathrm{CV}}$, $\gamma_{\mathrm{hip},\mathrm{ROM}}$
Knee	Sagittal	$\theta_{\mathrm{knee},\mathrm{Max}}$, $\theta_{\mathrm{knee},\mathrm{Min}}$, $\theta_{\mathrm{knee},\mathrm{maxTiming}}$, $\theta_{\mathrm{knee},\mathrm{minTiming}}$, $\theta_{\mathrm{knee},\mathrm{RMS}}$, $\theta_{\mathrm{knee},\mathrm{Mean}}$, $\theta_{\mathrm{knee},\mathrm{SD}}$, $\theta_{\mathrm{knee},\mathrm{CV}}$, $\theta_{\mathrm{knee},\mathrm{ROM}}$
	Coronal	$\beta_{\mathrm{knee},\mathrm{Max}}$, $\beta_{\mathrm{knee},\mathrm{Min}}$, $\beta_{\mathrm{knee},\mathrm{maxTiming}}$, $\beta_{\mathrm{knee},\mathrm{minTiming}}$, $\beta_{\mathrm{knee},\mathrm{RMS}}$, $\beta_{\mathrm{knee},\mathrm{Mean}}$, $\beta_{\mathrm{knee},\mathrm{SD}}$, $\beta_{\mathrm{knee},\mathrm{CV}}$, $\beta_{\mathrm{knee},\mathrm{ROM}}$
	Transverse	$\gamma_{\mathrm{knee},\mathrm{Max}}$, $\gamma_{\mathrm{knee},\mathrm{Min}}$, $\gamma_{\mathrm{knee},\mathrm{maxTiming}}$, $\gamma_{\mathrm{knee},\mathrm{minTiming}}$, $\gamma_{\mathrm{knee},\mathrm{RMS}}$, $\gamma_{\mathrm{knee},\mathrm{Mean}}$, $\gamma_{\mathrm{knee},\mathrm{SD}}$, $\gamma_{\mathrm{knee},\mathrm{CV}}$, $\gamma_{\mathrm{knee},\mathrm{ROM}}$
Ankle	Sagittal	$\theta_{\mathrm{ankle},\mathrm{Max}}$, $\theta_{\mathrm{ankle},\mathrm{Min}}$, $\theta_{\mathrm{ankle},\mathrm{maxTiming}}$, $\theta_{\mathrm{ankle},\mathrm{minTiming}}$, $\theta_{\mathrm{ankle},\mathrm{RMS}}$, $\theta_{\mathrm{ankle},\mathrm{Mean}}$, $\theta_{\mathrm{ankle},\mathrm{SD}}$, $\theta_{\mathrm{ankle},\mathrm{CV}}$, $\theta_{\mathrm{ankle},\mathrm{ROM}}$
	Coronal	$\beta_{\mathrm{ankle},\mathrm{Max}}$, $\beta_{\mathrm{ankle},\mathrm{Min}}$, $\beta_{\mathrm{ankle},\mathrm{maxTiming}}$, $\beta_{\mathrm{ankle},\mathrm{minTiming}}$, $\beta_{\mathrm{ankle},\mathrm{RMS}}$, $\beta_{\mathrm{ankle},\mathrm{Mean}}$, $\beta_{\mathrm{ankle},\mathrm{SD}}$, $\beta_{\mathrm{ankle},\mathrm{CV}}$, $\beta_{\mathrm{ankle},\mathrm{ROM}}$
	Transverse	$\gamma_{\mathrm{ankle},\mathrm{Max}}$, $\gamma_{\mathrm{ankle},\mathrm{Min}}$, $\gamma_{\mathrm{ankle},\mathrm{maxTiming}}$, $\gamma_{\mathrm{ankle},\mathrm{minTiming}}$, $\gamma_{\mathrm{ankle},\mathrm{RMS}}$, $\gamma_{\mathrm{ankle},\mathrm{Mean}}$, $\gamma_{\mathrm{ankle},\mathrm{SD}}$, $\gamma_{\mathrm{ankle},\mathrm{CV}}$, $\gamma_{\mathrm{ankle},\mathrm{ROM}}$

CV, coefficient of variation; Max, maximum; Min, minimum; RMS, root mean square; SD, standard deviation.

### 2.5 Machine learning model

Forward sequential feature selection in a wrapper fashion was used to select the optimal feature set from all the features. This approach begins with no features in the model and incrementally adds features based on their contribution to improving the accuracy of the classification model until the selection criteria are satisfied. The accuracy of the model was expressed as the misclassification rate, which was the number of misclassified samples as a percentage of the total number of samples (24). Five-fold cross-validation was adopted. The dataset was divided into five equally sized folds. When one fold was used as the test set, the remaining four folds formed the training set. The ML model was trained on the training set, and its accuracy was assessed by the test set. Five accuracy rates were obtained as each fold was used as the test set in turn. The average of the five accuracy rates was the final accuracy.

Support vector machine (SVM), *k*-nearest neighbors (KNN), naïve Bayes (NB), and decision trees (DT) models are commonly used in classification and prediction tasks (25). The accuracy of the four models was examined using leave-one-subject-out cross-validation (LOSOCV) based on the obtained optimal feature set (24). Similarly, each subject's data were used as the test set in turn, and the remaining data from the 15 subjects were the training set. The performance of each ML model was assessed by the average accuracy from the 16 tests. The loss function was the lowest misclassification cost. Bayesian optimization was used.

### 2.6 Statistical analysis

The sample size of 16 was calculated using G*Power 3.1.9.7 (Universität Düsseldorf, Düsseldorf, Germany) based on a significance level of 0.05, statistical power of 0.8, and a medium effect size of 0.34 using the within-factor *F*-test with three repeated measures. Since the data did not meet the hypothesis of the normal distribution test and the assumption of the homogeneity of variance, the statistical differences in features between the three groups of saddle heights were assessed using the Friedman test with a significance level of *α* < 0.05. A *post-hoc* pairwise comparison using the Wilcoxon signed-rank test with Bonferroni correction was conducted if significance was found in the Friedman test.

The classification accuracy rate based on a single feature in the optimal feature set was calculated by the ML model to characterize the contribution of each feature to the final performance of the model. The correlations between the individual features in the optimal set were measured using Pearson’s correlation coefficient, *r*, which was defined as a strong (|*r*| ≥ 0.7), moderate (0.5 ≤ |*r*| < 0.7), low (0.3 ≤ |*r*| < 0.5), or negligible correlation (|*r*| < 0.3) (26). The above analysis process, including processing the experimental data, dataset construction, feature extraction, calculation of model accuracy, and statistical analysis, was conducted in MATLAB R2024a (MathWorks Inc., Natick, Massachusetts, USA).

## 3 Results

### 3.1 Optimal feature set

**Figure 3:**
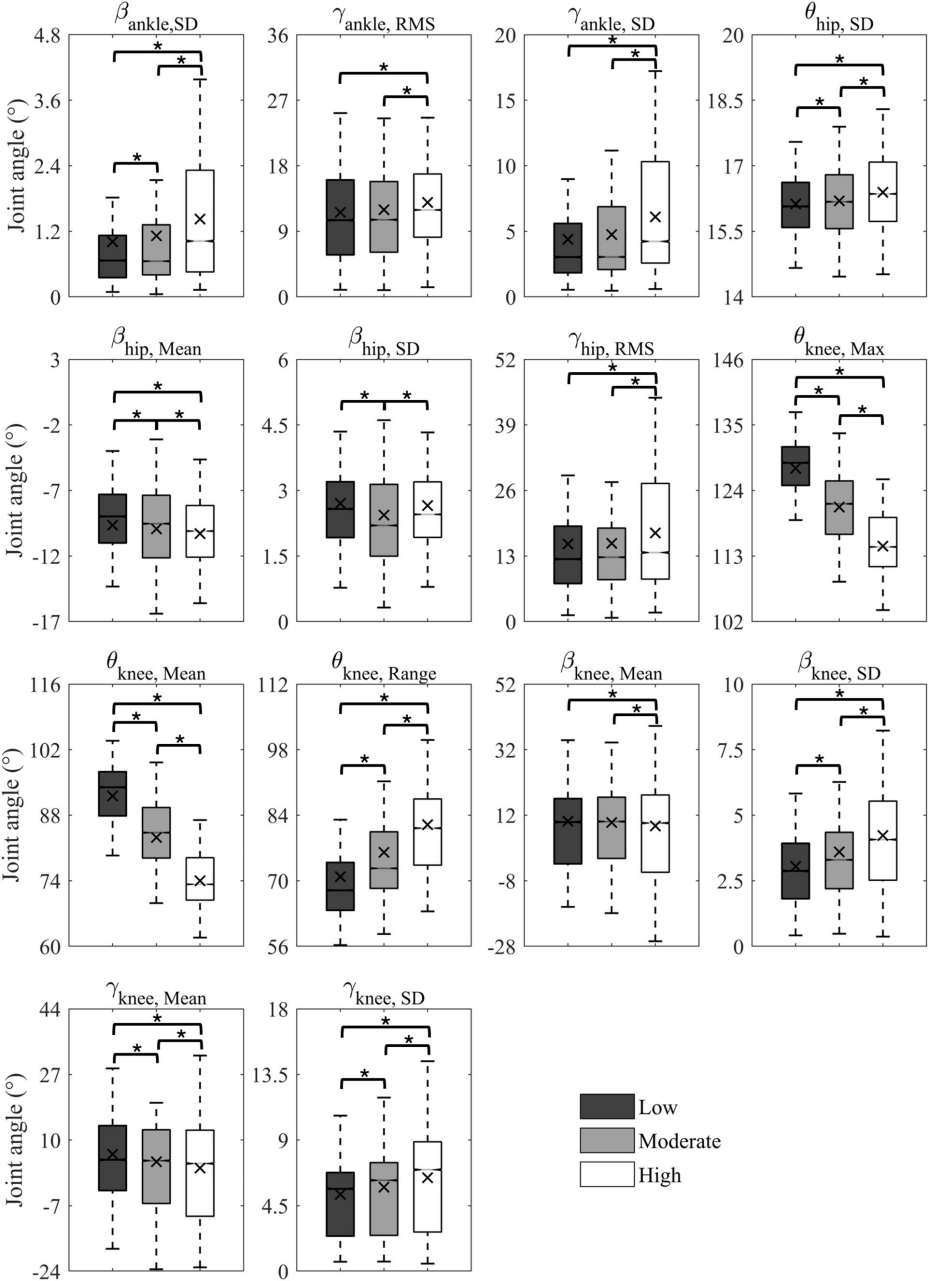
Statistical results of the selected features for the low, moderate, and high saddle heights.

**Figure 4:**
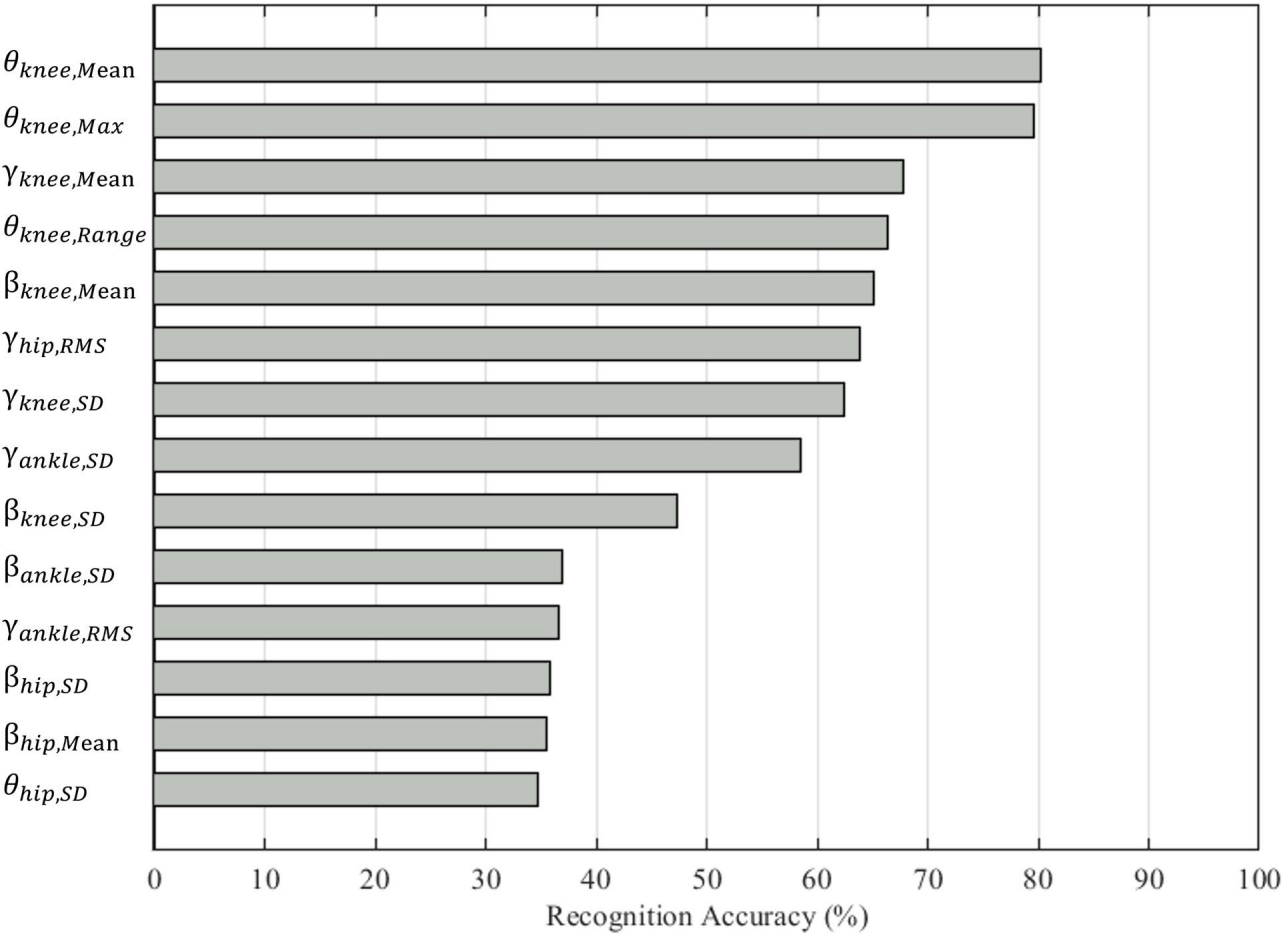
Classification accuracies based on individually selected features.

In total, 14 features were selected from 81 features to form the optimal set, including three ankle joint features ($\beta_{\mathrm{ankle},\mathrm{SD}}$, $\gamma_{\mathrm{ankle},\mathrm{RMS}}$, $\gamma_{\mathrm{ankle},\mathrm{SD}}$), four hip joint features ($\theta_{\mathrm{hip},\mathrm{SD}}$, $\beta_{\mathrm{hip},\mathrm{Mean}}$, $\beta_{\mathrm{hip},\mathrm{SD}}$, $\gamma_{\mathrm{hip},\mathrm{RMS}}$), and seven knee joint features ($\theta_{\mathrm{knee},\mathrm{Max}}$, $\theta_{\mathrm{knee},\mathrm{Mean}}$, $\theta_{\mathrm{knee},\mathrm{Range}}$, $\beta_{\mathrm{knee},\mathrm{Mean}}$, $\beta_{\mathrm{knee},\mathrm{SD}}$, $\gamma_{\mathrm{knee},\mathrm{Mean}}$, $\gamma_{\mathrm{knee}\;\mathrm{SD}}$). The selected features presented at least one set of statistical differences between the three saddle height levels, as shown in Figure 3. The most notable changes were in $\theta_{\mathrm{knee},\mathrm{Max}}$, $\theta_{\mathrm{knee},\mathrm{Mean}}$, $\theta_{\mathrm{knee},\mathrm{Range}}$, and $\beta_{\mathrm{knee},\mathrm{SD}}$ (*p* < 0.001) as the saddle height increased. $\beta_{\mathrm{knee},\mathrm{Mean}}$ was only statistically different between the low and high saddle height levels (*p* < 0.001). The classification accuracy of the saddle height levels based on one feature suggested a contribution of the selected feature to the final accuracy (Figure 4). $\theta_{\mathrm{knee},\mathrm{Mean}}$ and $\theta_{\mathrm{knee},\mathrm{Max}}$ achieved the highest accuracy rates of 80.19% and 79.58%, respectively. The top five features with the highest accuracy rankings were all related to knee joint angle. The bottom three features with the lowest accuracy rankings were $\beta_{\mathrm{hip},\mathrm{SD}}$, $\beta_{\mathrm{hip},\mathrm{Mean}}$, and $\theta_{\mathrm{hip},\mathrm{SD}}$, with accuracy rates around 35%.

**Figure 5:**
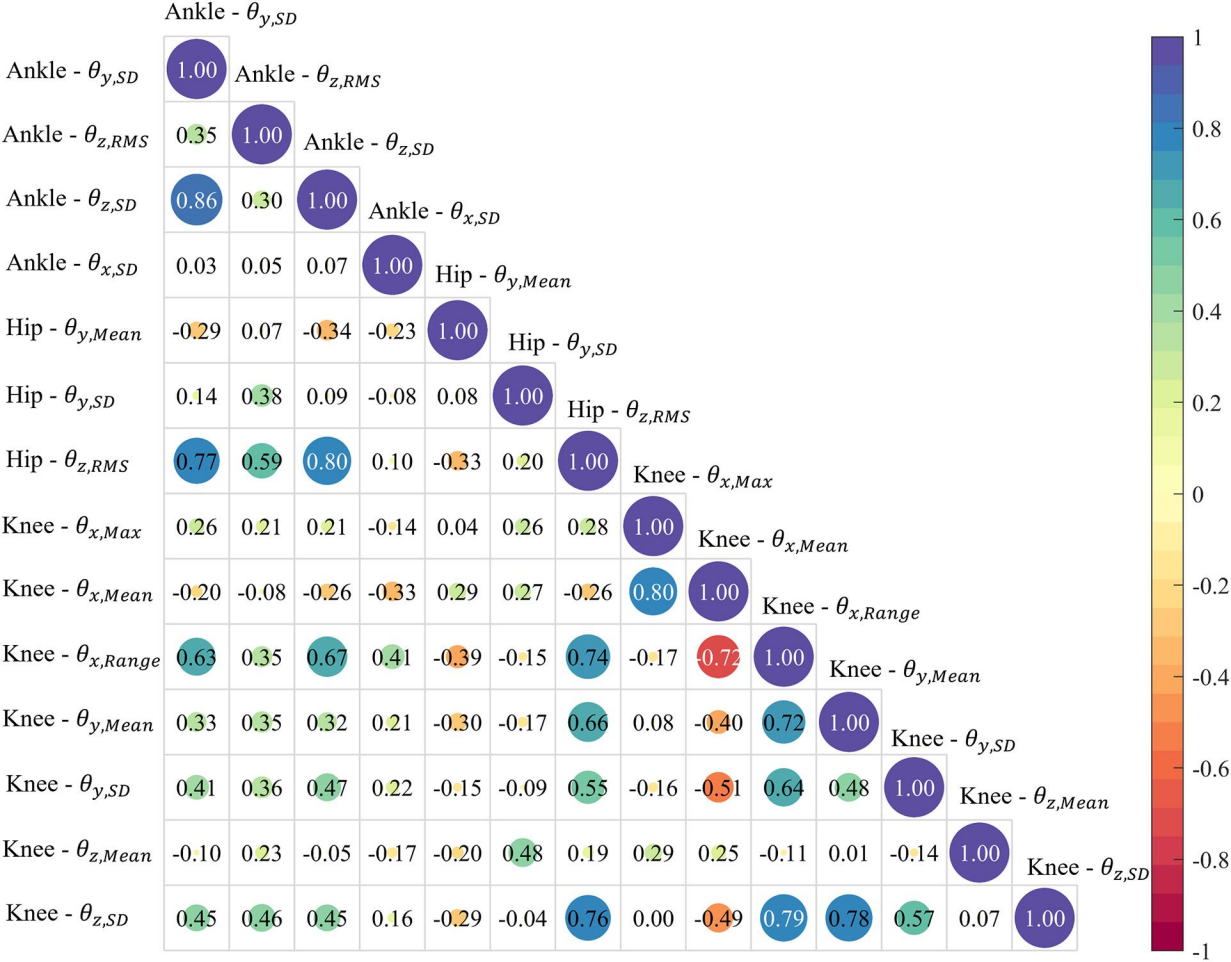
The correlation coefficients between the features in the optimal feature set.

Most of the correlation coefficients between features in the optimal feature set were less than 0.5, meaning that most of the features had a low or negligible correlation with each other (Figure 5). Strong correlations existed between $\beta_{\mathrm{ankle},\mathrm{SD}}$ and $\gamma_{\mathrm{ankle},\mathrm{SD}}$ (*r* = 0.86), $\gamma_{\mathrm{ankle},\mathrm{SD}}$ and $\gamma_{\mathrm{hip},\mathrm{RMS}}$ (*r* = 0.80), $\beta_{\mathrm{ankle},\mathrm{SD}}$ and $\gamma_{\mathrm{hip},\mathrm{RMS}}$ (*r* = 0.77), and $\theta_{\mathrm{knee},\mathrm{Max}}$ and $\theta_{\mathrm{knee},\mathrm{Mean}}$ (*r* = 0.77).

### 3.2 Comparison of machine learning models

**Figure 6:**
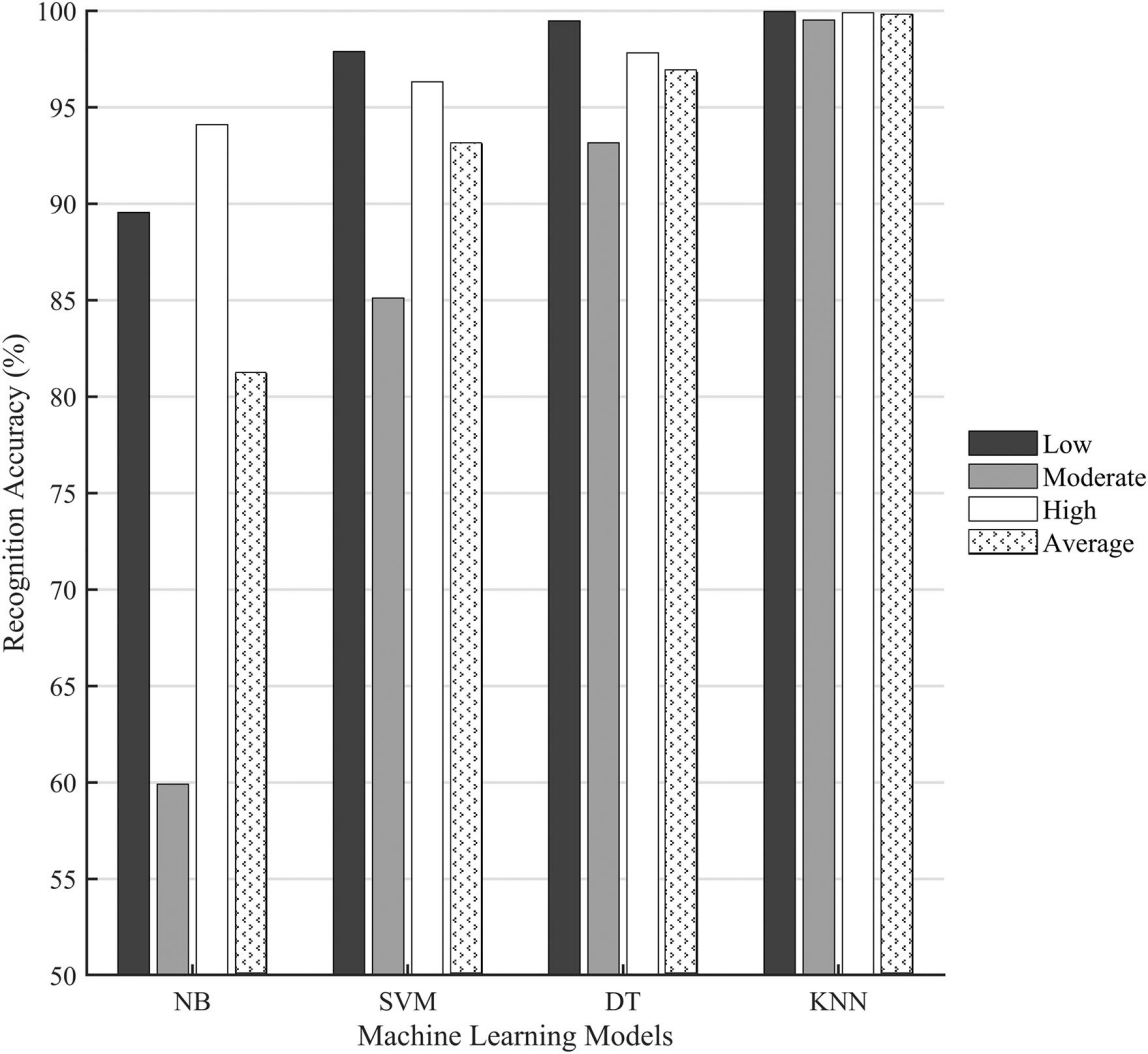
Comparison of the classification accuracies of four machine learning models. NB, Naïve Bayes; SVM, support vector machine; DT, decision tree; KNN, *k*-nearest neighbors.

The classification accuracies at each saddle height level and the averaged accuracies were compared among the SVM, KNN, NB, and DT models (Figure 6). The KNN model achieved the highest average accuracy of 99.79% and outperformed the other three models. It also performed the best in the classification of each saddle height level with an accuracy of 99.96% for the low level, 99.52% for the moderate level, and 99.89% for the high level. The DT model was a bit inferior to the KNN model, with an average accuracy of 96.81%, which was higher than that of the SVM and NB models. The DT model had the lowest classification accuracy for the moderate level (93.16%) and the highest for the low level (99.47%). The average accuracy of the SVM model was 93.10%. The NB model had the lowest average accuracy (81.18%) among the four models, especially for the moderate level, with the accuracy of only 59.91%.

## 4 Discussion

An improper saddle height can lead to knee strain, lower back pain, and reduced power output (2, 27). Conventional methods of optimizing saddle height have predominantly relied on static anthropometric indices, such as leg length (28). However, these methods exhibit significant limitations because they neglect the dynamic interactions within the lower limb kinematic chain and interindividual heterogeneity in biomechanical responses, which lead to different definitions of the optimal saddle height. For instance, a study demonstrated that the knee joint angles of only 37% of subjects were within the recommended range of 25°–35° when using the 109% inseam method to set the saddle height (29). Static and dynamic knee angles were found to be significantly different by approximately 8° at the BDC position (4). Such contradictions underscore the methodological inadequacy of static models in capturing real-time cycling kinematics. Despite the growing acceptance of dynamic measurements, the required equipment and techniques are not always available, especially for daily training and outdoor cycling. An ML model was developed in this study to classify the saddle height level based on features extracted from the dynamically measured angles of lower limb joints. This compensates for the limitations of existing ML models in bike fitting, as most models focus on predictions of a cyclist's physiological parameters and competition performance.

The statistical analysis of features revealed statistically significant variations in lower limb joint angles in three dimensions across the three saddle height levels (Figure 3). These multiplanar kinematic changes corroborate previous findings that a reduction in saddle height increases ankle dorsiflexion, flexion and abduction of the knee, and flexion of the hip, while the ROMs of three lower limb joints also decrease (30–32). Furthermore, the most significant variation has been observed in the flexion-extension knee joint angle features because the knee joint angle has the largest ROM in the sagittal plane. This was confirmed in a previous study, as there were more significant changes in knee flexion angle and its ROM than in ankle and hip joints during cycling with varied saddle heights in both cyclists and triathletes (30). A 5% increase in saddle height caused a 25% increase in the ROM of the knee flexion angle (33). This explains our results, as the mean, maximum, and range values of the sagittal plane knee angle varied most significantly at different saddle heights. A previous study also showed a notable increase in the ROM of the ankle dorsiflexion angle from 27° to 41° as saddle height increased (33). However, our optimal feature set only included the RMS and SD values associated with the abduction–adduction and inversion-eversion ankle angles. This discrepancy may result from the fact that we used Pyro Platform shoes with front and rear heel constraints in the experiment, whereas they used common commercial cycling shoes. In addition, the mean of the abduction–adduction angle and the RMS of the external–internal rotation angle of the hip joint were included in the optimal feature set. The changes in joint kinematics in the transverse and coronal planes revealed by ML analysis may provide new insights for future research, since most cycling studies have focused on the sagittal plane (34, 35).

The fact that the highest classification accuracy was based on a single knee flexion angle feature emphasizes the influence of saddle height on knee kinematics (Figure 4). A lower saddle height resulted in a decreased sagittal plane angle and decreased ROM of the knee and further induced a greater knee extension moment rather than an abduction moment (31). Furthermore, the abduction angle of the knee did not show significant changes in our study when the saddle height increased by less than 5%. The knee extension moment is an indicator of knee joint loading since it exhibits the same changing behavior at various saddle heights as the tibiofemoral compressive force (36). The SD values of the adduction/abduction and internal/external rotation angles of the knee in the optimal feature set increased at a higher saddle height, while their means declined. This may indicate that a high saddle height exacerbated oscillations and instability in the lower limbs. Therefore, saddle height adjustment is very important to prevent injuries to the knee joint.

The KNN model showed superior performance in saddle height classification compared to the other three ML models (Figure 6). However, all the models shared a common limitation: reduced accuracy in classifying the moderate saddle height level compared to the high and low levels. The NB model displayed particularly low accuracy of 59.91% for moderate heights. This may be related to the model's assumption of feature independence, but latent correlations always exist in human biomechanical datasets. Another critical reason is that the joint angles displayed more pronounced variations at extreme saddle heights. Cyclists may naturally exhibit greater movement variability when riding at moderate height deviations from their preferred position, as the biomechanical constraints are less severe than at extreme heights. This adaptive behavior increases intraclass variation for moderate conditions. The moderate height condition (97%–103% of GTH) encompassed a wider range of saddle heights compared to the singular high (105% GTH) and low (95% GTH) conditions. This introduced greater variance in joint angles and could reduce the model's ability to identify consistent patterns for classification. Moreover, the use of the synthetic minority oversampling technique increased the data volume for the high and low saddle height groups while maintaining the original sample size for the moderate height group, which created an imbalance in data dispersion. The high/low saddle heights showed a lower numerical variance compared to the moderate group, making their classification comparatively easier. Despite the dataset only including lower limb joint angle features, the low correlation coefficients shown in Figure 5 indicated that feature selection successfully isolated complementary and non-redundant predictors. However, strong correlations were found between a subset of features. While multicollinearity may have distorted the interpretability of the model, its impact was mitigated by two factors. First, most features exhibited low correlations, preserving the diversity of input information. Second, the top-performing KNN model is non-parametric and relies on distance metrics rather than coefficient weights, reducing sensitivity to inter-feature dependencies (37). In addition, the effects of saddle height on knee extension moments, oxygen uptake, and cycling efficiency have been identified (31, 38). Including more metrics, such as pedal force and power output, may enhance the model’s sensitivity to subtle saddle height differences.

It is difficult to compare the accuracy rates of our model with other studies because we could not find similar ML models that identified saddle heights during dynamic cycling. However, the developed KNN model in this study achieved a high accuracy of 99.79%, which already demonstrated its superiority and the effectiveness of the approach. Several ML models have been developed to recognize cycling parameters such as cadence (16) and pedaling profiles (39) with high accuracies of more than 95%. Their data were acquired by inertial measurement units (IMUs). It has been reported that IMU measurement of joint angles has a lower error rate per pedaling cycle (40). Therefore, IMUs can be used instead of motion capture systems to measure joint angles in future studies, enabling real-world outdoor cycling experiments.

By utilizing lower limb joint angle features as inputs, the developed model achieved high saddle height classification accuracy, providing a more objective and personalized approach by considering the dynamic effects in cycling. However, this study still has the following limitations. First, the recruited cyclists were not stratified by gender, age, or skill level, which could potentially affect the generalizability of the model. Gender disparity has been demonstrated in previous studies (41, 42), but it was neglected in this study. Due to differences in anthropometry, such as leg length and segment mass distribution, men and women may have different joint angles at the same saddle height. This may increase the dispersion of the original data used to train the model and subsequently affect the classification accuracy of the model. Second, the data were collected by a motion capture system and there is a discrepancy between cycling in the lab and actual outdoor cycling. Third, each feature in the optimal set was selected and verified, but some pairs of features still exhibited strong correlations. Future studies should classify participants based on their gender and cycling skills and use portable sensors, such as IMUs, to collect data during outdoor cycling (43). The accuracy and universality of the ML model can be improved by incorporating more kinematic and kinetic variables. Various ML models should be constructed for bike fitting for other configurations and cycling disciplines (e.g., road cycling vs. mountain biking).

## 5 Conclusion

This study developed a KNN machine learning model that had high accuracy when identifying saddle height levels using lower limb joint angle features. The four evaluated ML models showed lower accuracy for the moderate saddle height level compared to the low and high levels. The redundancy of the inputs and the correlations between the features were reduced by screening the optimal feature set. The sagittal plane knee joint angle was the variable most sensitive to saddle height, with a classification accuracy of 80.19% based on this feature. When the ankle and hip joint angles were included, the classification accuracy was improved to 99.79%. This approach highlights the potential for leveraging data-driven tools in cycling to provide personalized bike fitting and objective recommendations.

## Data Availability

The raw data supporting the conclusions of this article will be made available by the authors, without undue reservation.
